# A Privacy-Preserved ID-Based Secure Communication Scheme in 5G-IoT Telemedicine Systems

**DOI:** 10.3390/s22186838

**Published:** 2022-09-09

**Authors:** Tzu-Wei Lin

**Affiliations:** 1 i. School, Feng Chia University, Taichung 402, Taiwan; tweilin@fcu.edu.tw; 2 Information Security Office, Office of Information Technology, Feng Chia University, Taichung 402, Taiwan

**Keywords:** telemedicine systems, 5G, IoT, emergency medical services, privacy preservation

## Abstract

5G networks have an efficient effect in providing quality of experience and massive Internet of things (IoT) communication. Applications of 5G-IoT networks have been expanded rapidly, including in smart medical healthcare. Emergency medical services (EMS) hold an assignable proportion in our lives, which has become a complex network of all types of professionals, including care in an ambulance. A 5G network with EMS can simplify the medical treatment process and improve the efficiency of patient treatment. The importance of healthcare-related privacy preservation is rising. If the work of privacy preservation fails, not only will medical institutes have economic and credibility losses but also property losses and even the lives of patients will be harmed. This paper proposes a privacy-preserved ID-based secure communication scheme in 5G-IoT telemedicine systems that can achieve the features below. (i) The proposed scheme is the first scheme that integrates the process of telemedicine systems and EMS; (ii) the proposed scheme allows emergency signals to be transmitted immediately with decreasing risk of secret key leakage; (iii) the information of the patient and their prehospital treatments can be transmitted securely while transferring the patient to the destination medical institute; (iv) the quality of healthcare services can be assured while preserving the privacy of the patient; (v) the proposed scheme supports not only normal situations but also emergencies. (vi) the proposed scheme can resist potential attacks.

## 1. Introduction

The 5G (fifth generation) networks are the newest standard of mobile telecommunication that is being deployed on the earth. 5G networks provide speed, capacity, and scalability, which have an efficient effect on energy consumption and provide quality of services (QoS) and amount of devices communication [1,2]. A device connects with a small base station through high-band spectrum technology and devices-to-devices communication [1,3,4]. 5G networks combine and connect virtual systems to the cloud and help derive different calculating models [5]. 5G networks will have a huge impact on connected services and devices through higher reliability, connectivity, and storage [5]. Internet of things (IoT) arranges objects as a part of network settings in a distributed network. IoT has become a concept of enclosing several technologies and a network between objects and human beings, which can interact and cooperate with other devices to communicate and share information. The vision of next-generation 5G wireless communications lies in providing very high data rates, extremely low latency, manifold an increase in base station capacity, and significant improvement in users’ perceived quality of service compared to current 4G LTE networks [6]. 5G can significantly increase the capacity and speed to provide reliable and speedy connectivity to the future IoT and, moreover, provide reliable connections to thousands of devices at the same time [7]. 5G will be able to provide a massive connection of Internet of things (IoT), where billions of smart devices can be connected to the internet [7]. However, security and privacy issues of transmitted information between objects are rising these years because wireless communications are vulnerable to many adversarial attacks, which is an important transmitting media of IoT networks.

Medical healthcare systems currently have many challenges, such as infrastructure, connections, professional requirements, data management, real-time monitoring, etc., and each challenge affects the quality of healthcare services [8]. Applications of 5G networks have been expanded rapidly, including in healthcare, and IoT with 5G environments provides solutions for network layers, including enhancing QoS to solve the challenges above [1,4]. On the other hand, the importance of healthcare-related privacy preservation is rising. If the work of privacy preservation fails, not only will medical institutes have economic and credibility losses but also property losses and even the lives of patients will be harmed. Maintaining the privacy of patient data, which is usually stored in conventional systems and difficult to share due to varying standards and data formats, is one of the important sectors of the healthcare industry. If the healthcare information of patients is the key to finding medical treatment, maintaining the privacy of patient data becomes a central issue that determines the success of medical practices [8].

Emergency medical services (EMS) hold an assignable proportion in our lives, which has become a complex network of all types of professionals, including care in an ambulance, serving as educators, practicing community paramedicine, and conducting research [9]. EMS has to be the first to respond and take care of minor and major injured patients while attending to calls coming from different situations, such as accidents, natural disasters, terrorism, pandemics, and patient transport. The state of California Emergency Medical Services Authority of US developed a search, alert, file, and reconcile (SAFR) model to reach goals of bidirectional data exchange between the EMS and the health information exchange (HIE) organization to enhance prehospital treatments, prehospital decision-making, better longitudinal patient record, and overall care [10]. The 5G network has the potential to bring benefits to individuals, organizations, and society, which enables ambulances to connect a patient who wears wearable devices to the emergency department of the destination hospital. Measured biodata is collected at the incident scene and transmitted to the servers of the destination hospital when the patient is being transported, which can allow the medical professional team at the destination hospital to immediately realize the condition of the patient, the prehospital treatment performed by a medical professional on an ambulance, and help decision-making. Measured biodata can be interconnected with hospital information systems, laboratory information systems, geographic information systems, picture archiving and communications systems, and document management systems, which enable medical professionals in destination hospitals to realize the historical medical records of patients, decide first-aid information, and issue examination sheets. 5G networks with EMS can simplify the medical treatment process and improve the efficiency of patient treatment [11].

This paper proposes a privacy-preserved ID-based secure communication scheme in 5G-IoT telemedicine systems that can achieve the features below. (i) The proposed scheme allows emergency signals to be transmitted immediately with decreasing risk of secret key leakage; (ii) the information of the patient and their prehospital treatments can be securely transmitted while transferring the patient to the destination medical institute; (iii) the quality of healthcare services can be assured while preserving the privacy of the patient; (iv) the proposed scheme supports not only normal situations but also emergencies. (v) the proposed scheme can resist potential attacks. The remaining organization of paper is sketched below. Telemedicine systems, federal identity management mechanisms, key insulation, and Chebyshev chaotic maps are introduced in Section 2. Section 3 introduces the proposed scheme, and security and performance analysis are detailed in Section 4 and Section 5. Finally, the conclusion is drawn in Section 6.

## 2. Related Works

Telemedicine systems are a combination of healthcare, electronic messaging, and telecommunication technology [8,12,13]. Patients can transmit healthcare-related information, which is usually important, sensitive, and private, to healthcare services through public networks when using telemedicine systems [8,12,13]. This means that medical professionals are able to know the health condition of a patient immediately and following up on the health condition of the patient becomes more convenient than before [12]. A general telemedicine system in 5G-IoT environments includes three types of telemedicine, which are synchronous telemedicine, asynchronous telemedicine, and remote health monitoring [2,14]. Synchronous telemedicine allows the patient and the medical professional to communicate directly through telecommunication technology, such as Microsoft Teams (version 1.5, Microsoft Corporation, Washington, US), Cisco Webex (version 42.9, Cisco Systems, San Jose, California, US), Zoom (version 5.11, Zoom Video Communications, Inc., San Jose, California, US), etc. Asynchronous telemedicine means that the medical professional can follow up on the patient’s health condition through biodata continually transmitted by the patient and stored and analyzed by the server in the medical institute. Furthermore, the system can automatically notify the medical professional when the patient’s health condition turns bad after analyzing and predicting the biodata. Remote health monitoring allows the medical professional in real-time to monitor the patient’s health condition, and the medical professional can receive an alert immediately if an emergency happens to the patient through this type of telemedicine. This paper focuses on the scenarios of remote health monitoring and asynchronous telemedicine. Meanwhile, data transmission security will be discussed, such as eavesdropping, man-in-the-middle (MITM) attack, data tempering attack, message modification attack, data interception attack, etc. [8,15]. Technical support is not enough though famous regulations providing personal information privacy have been announced [8,15].

Shamir introduced an identity-based (ID-based) cryptosystem [16], and an ID-based cryptosystem derives the user’s public key from the public and unique information of the user. Gentry et al. developed hierarchical ID-based cryptography (HIDC) based on the original ID-based cryptosystem, and HIDC has been proven to reduce the loading of private key generation and the risk of key escrow [17]. Several works have been proposed in the past two decades [18,19,20,21], including Santos et al.’s work, which is a lightweight federal identity management mechanism for IoT [22]. Moreover, Lin and Hsu [8] proposed a hierarchical ID-based cryptography for federal identity management in telemedicine in a 5G-IoT environment, which includes IoT gateways in the system structure. The proposed scheme applied a similar structure that the smart lamp replaces IoT gateway in the work of Lin and Hsu [8], and the scenario of the proposed scheme includes an emergency that is not included in Lin and Hsu’s work [8]. 

Key insulation, which is introduced by Dodis et al., is one of the effective solutions to a key exposure problem [23]. More and more wearable healthcare devices are used, and they only have limited resources to protect keys. Any malicious adversary can easily obtain the key information of users or devices, which leads to the key exposure problem. Once a private key is compromised, a malicious adversary has the chance to use the exposed private key to submit a legitimate request [24]. In a public key cryptosystem that is key-insulated, a receiver has two types of secret keys, a decryption key and a helper key. The decryption key is a short-term key for decrypting ciphertexts and is periodically updated by the helper key. More specifically, the lifetime of a system is divided into discrete time periods, and the receiver can decrypt the ciphertext, which is encrypted at some time period, by using a decryption key updated by the helper key at the same time period. The decryption key is stored in a powerful but insecure device such as portable healthcare devices, and the helper key is stored in a physically secure but computationally limited device called a helper, such as a smartphone. Key-insulated encryption can significantly reduce the impact of the key exposure problem, and many researchers have taken several approaches to realize secure key-insulated cryptosystems. Many cryptographers have proposed several types of key-insulated cryptographic schemes, such as symmetric-key-based key-insulated encryption [23], key-insulated signatures [25], parallel key-insulated encryption [24,26], etc.

A chaotic system has features that can correspond to important features, confusion and diffusion of cryptosystems [27,28,29]. First, the result of a chaotic system is unpredictable if small changes in initial values happen [27,30]. Second, a chaotic system is a complex oscillation [27,30]. Third, a chaotic system has a qualitative change of character of solutions [27,30]. Cryptosystems based on Chebyshev chaotic maps have been widely discussed for decades, including lightweight solutions [13,28,29,31,32,33]. Mathematical definitions of Chebyshev chaotic maps are given in Table 1 [13,28,29,31,32,33]. Proposed schemes in this paper apply extended Chebyshev chaotic maps that satisfy definitions in Table 1.

## 3. Proposed Scheme

**Figure 1:**
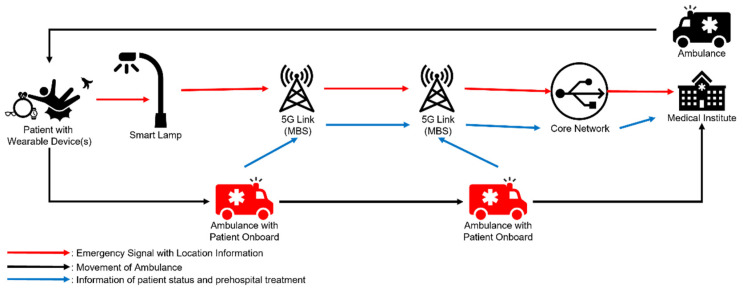
System structure of proposed scheme.

In this paper, a scenario that includes a patient ${Pa}_{i}$, a smart lamp ${SL}_{j}$, an ambulance $A_{ij}$, and a server of a medical institute (*MS*) is focused as illustrated in Figure 1. 

**Figure 2:**
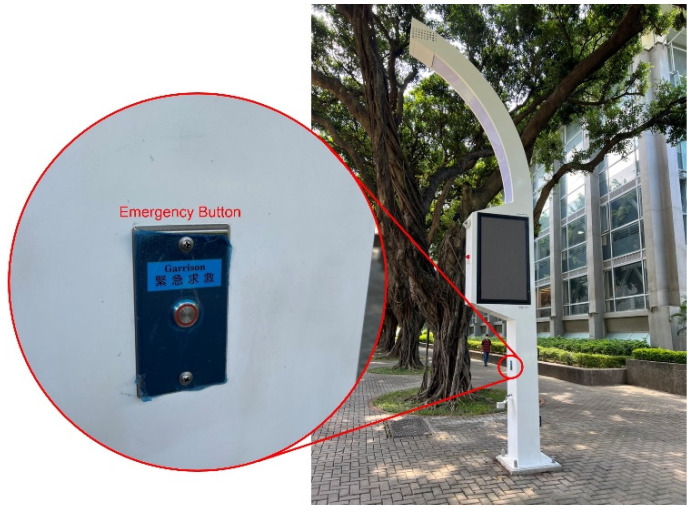
Smart lamp with emergency button.

Once an emergency occurs to the patient, an emergency signal is sent by the wearable device(s) to a nearby smart lamp, and then the smart lamp transmits a signal to the nearest medical institute. Another way for the smart lamp to send an emergency signal is for other passersby nearby the patient to press the emergency button on the smart lamp, as in Figure 2. After receiving the signal, a medical institute resolves the location of the patient, transmits related information to EMS staff, and assigns an ambulance to the site. After EMS staff move the patient into the ambulance, EMS can send information about the patient, including status and prehospital treatments to the destination medical institute. The staff of the emergency department at the destination medical institute can provide proper treatment according to the information on the prehospital treatments after receiving the patient. The interaction between 5G links and a core network should be secure, which may be guaranteed by functions in the core network, but secure communication between 5G links and a core network is not discussed in the proposed scheme.

The proposed scheme has five phases: system initialization phase, registration phase, key update phase, emergency signal sending phase, and secure ambulance communication phase. In the system initialization phase, the server of the medical institute (*MS*) generates essential parameters and functions. The patient (${Pa}_{i}$), smart lamp (${SL}_{j}$), and ambulance ($A_{ij}$) become legitimate parties through a registration phase. In the key update phase, a patient’s (${Pa}_{i}$’s) smartphone can help a patient (${Pa}_{i}$) update keys and secure a component in the smart lamp that can help the smart lamp (${SL}_{j}$) update keys. In the secure ambulance communication phase, the ambulance ($A_{ij}$) and the smart lamp (${SL}_{j}$) authenticate each other and establish a session key for symmetric encryption for communication and transmitted information on the status and prehospital treatments. Notations are defined in Table 2.

### 3.1. System Initialization Phase

In the system initialization phase, a server of a medical institute (*MS*), which provides telemedicine services and is certified by a healthcare certification authority, sets up parameters by performing the following steps.

Step 1: The healthcare certification authority issues a certificate ${\mathrm{Certificate}}_{\mathrm{HCA}\to \mathrm{MS}}$ to the server of a medical institute (*MS*) that provides telemedicine services and is certified by a healthcare certification authority.

Step 2: The server of a medical institute (*MS*) generates secret values ($s_{\mathrm{MS}}{,\;\omega }_{\mathrm{MS}})\in Z_{p}^{*}$, a big prime *p*, and a random number *x*∈(−∞,+∞) and computes $P_{\mathrm{MS}}$ and $P_{\mathrm{HA}}$ according to mathematical definitions of extended Chebyshev polynomials in Table 1.

(1) $$P_{\mathrm{MS}}=T_{s_{\mathrm{MS}}}(x)\;\mathrm{mod}\;p$$

(2) $$P_{\mathrm{HA}}=T_{\omega_{\mathrm{MS}}}(x)\;\mathrm{mod}\;p$$

Step 3: The server of a medical institute (*MS*) chooses a symmetric encryption algorithm $E_{k}(.)$, a symmetric decryption algorithm $D_{k}(.)$, collision-resistance one-way hash functions ($H_{0}$(.), $H_{1}$(.), $H_{2}$(.)) where $H∶{\left\{0,\;1\right\}}^{*}\to {\left\{0,\;1\right\}}^{n}$ that takes a binary string *q* $\in {\left\{0,\;1\right\}}^{*}$ of any arbitrary length as input and produces a binary string $H_{q}\in {\left\{0,\;1\right\}}^{n}$ as an output, and a collision-resistance secure one-way chaotic keyed hash function $h_{k}$(.).

Step 4: The server of a medical institute (*MS*) outputs public parameters ${\{P}_{\mathrm{MS}}{,\;P}_{\mathrm{HA}}{,\;p,\;x,\;H}_{0}({.),\;H}_{1}({.),\;H}_{2}({.),\;h}_{k}({.),\;E}_{k}({.),\;D}_{k}(.)\}$ and private parameters ($s_{\mathrm{MS}}{,\;\omega }_{\mathrm{MS}})$.

Step 5: The smart lamp (${\mathrm{SL}}_{j}$) generates two large random primes ($p_{j}$, $q_{j}$), and $\varphi_{j}$. Then, the smart lamp (${\mathrm{SL}}_{j}$) selects a random integer $e_{j}$, where $1\;<\;e_{j}\;<\;\varphi_{j}$ and gcd($e_{j}$, $\varphi_{j}$) = 1, and makes it public. After that, the smart lamp (${\mathrm{SL}}_{j}$) computes $d_{j}$, where ${1\;<\;d}_{j}\;<\;\varphi_{j}$ and $e_{j}d_{j}\;\equiv \;1\;(\mathrm{mod}\;\varphi_{j})$ and keeps $d_{j}$ secretly.

### 3.2. Registration Phase

**Figure 3:**
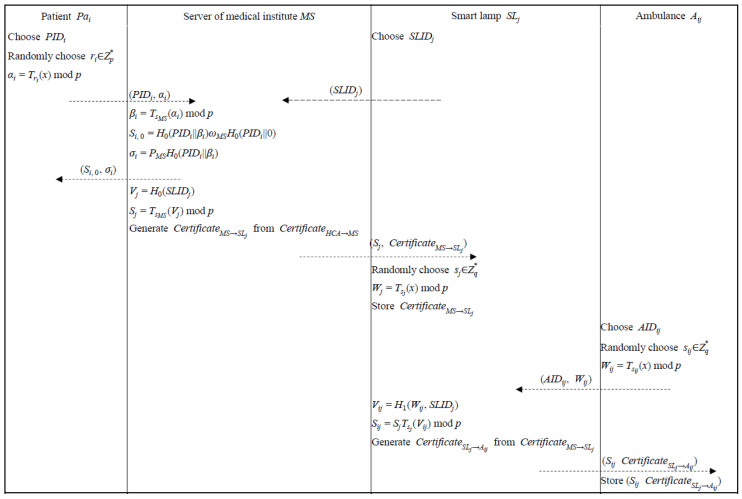
Registration phase.

In this phase, the patient (${\mathrm{Pa}}_{i}$) and the smart lamp (${\mathrm{SL}}_{j}$) interact with the server of a medical institute (*MS*) for registration, and the ambulance ($A_{\mathrm{ij}}$) interacts with the smart lamp (${\mathrm{SL}}_{j}$) for registration via a secure channel. To deal with the registration request submitted by the patient (${\mathrm{Pa}}_{i}$) and the smart lamp (${\mathrm{SL}}_{j}$), the server of a medical institute (*MS*) validates the legitimacy of the patient ${\mathrm{Pa}}_{I}$ and the smart lamp ${\mathrm{SL}}_{j}$. After that, the server of a medical institute (*MS*) issues a private key ($S_{j}$) and a certificate ${\mathrm{Certificate}}_{\mathrm{MS}\to {\mathrm{SL}}_{j}}$ via a secure channel while computing and sending $\sigma_{I}$ to the patient (${\mathrm{Pa}}_{i}$). The ambulance ($A_{\mathrm{ij}}$) submits registration information to the smart lamp (${\mathrm{SL}}_{j}$), and the smart lamp (${\mathrm{SL}}_{j}$) verifies the ambulance’s ($A_{\mathrm{ij}}$) legitimacy then issues private key ($S_{\mathrm{ij}}$) and certificate ${\mathrm{Certificate}}_{{\mathrm{SL}}_{j}\to A_{\mathrm{ij}}}$. Detailed descriptions are stated as follows and illustrated in Figure 3.

Step 1: The patient, ${\mathrm{Pa}}_{i}$, chooses an identifier, ${\mathrm{PID}}_{i}$, and a random number, $r_{i}\in Z_{p}^{*}$, and computes $\alpha_{i}$. After that, the patient, ${\mathrm{Pa}}_{i}$, sends (${\mathrm{PID}}_{i}{,\;\alpha }_{i}$) to the server of a medical institute (*MS)*. Meanwhile, the smart lamp, ${\mathrm{SL}}_{j}$, chooses an identifier, ${\mathrm{SLID}}_{j}$, and submits to the server of a medical institute (*MS*).

(3) $$\alpha_{i}{=T}_{r_{i}}(x)\;\mathrm{mod}\;p$$

Step 2: After receiving (${\mathrm{PID}}_{i}{,\;\alpha }_{i}$) from the patient (${\mathrm{Pa}}_{i}$) and ${\mathrm{SLID}}_{j}$ from the smart lamp (${\mathrm{SL}}_{j}$), the server of a medical institute (*MS*) computes the elements below. Then, the server of a medical institute (*MS*) returns ($S_{i,\;0},\;\sigma_{i}$) to the patient (${\mathrm{Pa}}_{i}$) and $S_{j}$ with ${\mathrm{Certificate}}_{\mathrm{MS}\to {\mathrm{SL}}_{j}}$, which is generated by the server of a medical institute (*MS*), to the smart lamp (${\mathrm{SL}}_{j}$).

(4) $$\beta_{i}{=T}_{s_{\mathrm{MS}}}(\alpha_{i})\;\mathrm{mod}\;p$$

(5) $$S_{i,\;0}{=H}_{0}{(\mathrm{PID}}_{i}\left|\right|\beta_{i}{)\omega }_{\mathrm{MS}}H_{0}{(\mathrm{PID}}_{i}\left||0\right)$$

(6) $$\sigma_{i}{=P}_{\mathrm{MS}}H_{0}{(\mathrm{PID}}_{i}\left|\right|\beta_{i})$$

(7) $$V_{j}{=H}_{0}({\mathrm{SLID}}_{j})$$

(8) $$S_{j}{=T}_{s_{\mathrm{MS}}}{(V}_{j})\;\mathrm{mod}\;p$$

Step 3: The smart lamp (${\mathrm{SL}}_{j}$) chooses a random number $s_{j}\in Z_{q}^{*}$ as a secret value and computed $W_{j}$ and stores ${\mathrm{Certificate}}_{\mathrm{MS}\to {\mathrm{SL}}_{j}}$.

(9) $$W_{j}{=T}_{s_{j}}(x)\;\mathrm{mod}\;p$$

Step 4: The ambulance ($A_{\mathrm{ij}}$) chooses an identifier (${\mathrm{AID}}_{\mathrm{ij}}$) and a random number ($s_{\mathrm{ij}}\in {\;Z}_{p}^{*}$) as a secret value, computes $W_{\mathrm{ij}}$, and sends (${\mathrm{AID}}_{\mathrm{ij}}$, $W_{\mathrm{ij}}$) to the smart lamp (${\mathrm{SL}}_{j}$).

(10) $$W_{\mathrm{ij}}{=T}_{s_{\mathrm{ij}}}(x)\;\mathrm{mod}\;p$$

Step 5: After receiving ${\mathrm{AID}}_{\mathrm{ij}}$ from the ambulance ($A_{\mathrm{ij}}$), the smart lamp (${\mathrm{SL}}_{j}$) checks the format of ${\mathrm{AID}}_{\mathrm{ij}}$. If ${\mathrm{AID}}_{\mathrm{ij}}$ is valid, the smart lamp ${\mathrm{SL}}_{j}$ computes a private key $S_{\mathrm{ij}}$ corresponding to the ${\mathrm{AID}}_{\mathrm{ij}}$, then generates the ${\mathrm{Certificate}}_{{\mathrm{SL}}_{j}\to A_{\mathrm{ij}}}$ from the ${\mathrm{Certificate}}_{\mathrm{MS}\to {\mathrm{SL}}_{j}}$, and sends ($S_{\mathrm{ij}}$, ${\mathrm{Certificate}}_{{\mathrm{SL}}_{j}\to A_{\mathrm{ij}}}$) to the ambulance ($A_{\mathrm{ij}}$) via a secure channel.

(11) $$V_{\mathrm{ij}}{=H}_{1}(W_{\mathrm{ij}}{,\;\mathrm{SLID}}_{j})$$

(12) $$S_{\mathrm{ij}}{=S}_{j}T_{s_{j}}(V_{\mathrm{ij}})\;\mathrm{mod}\;p$$

Step 6: The ambulance ($A_{\mathrm{ij}}$) stores ($S_{\mathrm{ij}}$, ${\mathrm{Certificate}}_{{\mathrm{SL}}_{j}\to A_{\mathrm{ij}}}$).

### 3.3. Key Update Phase

**Figure 4:**
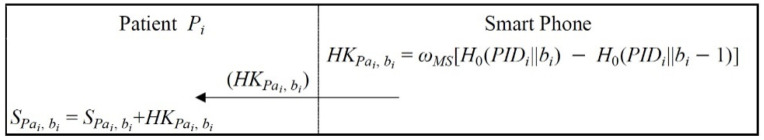
Key update phase.

The patient’s (${\mathrm{Pa}}_{i}$’s) smartphone can help the patient (${\mathrm{Pa}}_{i}$) update keys through following the steps as illustrated in Figure 4.

Step 1: The smartphone computes and sends the helper key ${\mathrm{HK}}_{{\mathrm{Pa}}_{i}{,\;b}_{i}}$ as below.

(13) $${\mathrm{HK}}_{{\mathrm{Pa}}_{i}{,\;b}_{i}}{=\omega }_{\mathrm{MS}}{[H}_{0}{(\mathrm{PID}}_{i}{||b}_{i})\;-{\;H}_{0}{(\mathrm{PID}}_{i}{||b}_{i}-1)]$$

Step 2: After receiving ${\mathrm{HK}}_{{\mathrm{Pa}}_{i}{,\;b}_{i}}$, the patient (${\mathrm{Pa}}_{i}$) computes $S_{{\mathrm{Pa}}_{i}{,\;b}_{i}}$ to update the key.

(14) $$S_{{\mathrm{Pa}}_{i}{,\;b}_{i}}{=S}_{{\mathrm{Pa}}_{i}{,\;b}_{i}}{+\mathrm{HK}}_{{\mathrm{Pa}}_{i}{,\;b}_{i}}$$

### 3.4. Emergency Signal Sending Phase

**Figure 5:**
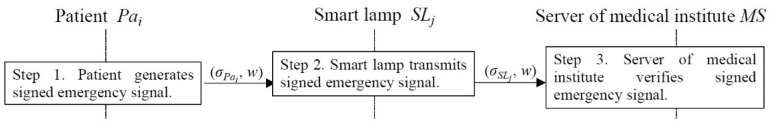
Emergency signal sending phase.

When an emergency happens to a patient (${\mathrm{Pa}}_{i}$) outdoors, the patient (${\mathrm{Pa}}_{i}$) can commission a nearby smart lamp (${\mathrm{SL}}_{j}$) to sign and send an emergency signal (${\mathrm{EM}}_{i}$) to a server of a medical institute (*MS*). The server of the medical institute (*MS*) can verify the message from patient (${\mathrm{Pa}}_{i}$) through the following steps as illustrated in Figure 5.

Step 1: The patient generates a signed emergency signal. The patient (${\mathrm{Pa}}_{i}$) computes ($\sigma_{{\mathrm{Pa}}_{i}1}$, $\sigma_{{\mathrm{Pa}}_{i}2}$) as below and sends ($\sigma_{{\mathrm{Pa}}_{i}},\;w$) to the smart lamp (${\mathrm{SL}}_{j}$) that *w* is a warrant including delegation information generated by patient (${\mathrm{Pa}}_{i}$).

(15) $$\sigma_{{\mathrm{Pa}}_{i}1}{=S}_{{\mathrm{Pa}}_{i}{,\;b}_{i}}r_{i}H_{1}{(\mathrm{EM}}_{i})$$

(16) $$\sigma_{{\mathrm{Pa}}_{i}2}{=\alpha }_{i}$$

(17) $$\sigma_{{\mathrm{Pa}}_{i}}{=(\sigma }_{{\mathrm{Pa}}_{i}1}{,\;\sigma }_{{\mathrm{Pa}}_{i}2}{,\;\mathrm{EM}}_{i}{,\;b}_{i})$$

Step 2: The smart lamp transmits a signed emergency signal. After receiving ($\sigma_{{\mathrm{Pa}}_{i}},\;w$), the smart lamp (${\mathrm{SL}}_{j}$) computes ($\sigma_{{\mathrm{SL}}_{j}1}$, $\sigma_{{\mathrm{SL}}_{j}2}$, $\sigma_{{\mathrm{SL}}_{j}3}$) as below and sends ($\sigma_{{\mathrm{SL}}_{j}},\;w$) to the server of the medical institute (*MS*).

(18) $$\sigma_{{\mathrm{SL}}_{j}1}{=\sigma }_{{\mathrm{Pa}}_{i}1}S_{{\mathrm{SL}}_{j}{,\;b}_{j}}r_{i}H_{2}{(\mathrm{EM}}_{i}{)r}_{i}H_{1}\left(w\right)$$

(19) $$\sigma_{{\mathrm{SL}}_{j}2}{=\sigma }_{{\mathrm{Pa}}_{i}2}\alpha_{i}$$

(20) $$\sigma_{{\mathrm{SL}}_{j}3}{=\alpha }_{i}$$

(21) $$\sigma_{{\mathrm{SL}}_{j}}{=(\sigma }_{{\mathrm{SL}}_{j}1},\;\sigma_{{\mathrm{SL}}_{j}2},\;\sigma_{{\mathrm{SL}}_{j}3}{,\;\mathrm{EM}}_{i}{,\;b}_{i}{,\;b}_{j})$$

Step 3: The server of the medical institute verifies the signed emergency signal. After receiving ($\sigma_{{\mathrm{SL}}_{j}},\;w$), the server of the medical institute (*MS*) verifies the message as below. If it holds, the server of the medical institute (*MS*) can confirm that the message was sent from the patient (${\mathrm{Pa}}_{i}$). The server of the medical institute (*MS*) utilizes information from the smart lamp ${(\sigma }_{{\mathrm{SL}}_{j}1}$, $\sigma_{{\mathrm{SL}}_{j}2}$, $\sigma_{{\mathrm{SL}}_{j}3}{,\;\mathrm{EM}}_{i}{,\;b}_{j})$ to compute verification parameters ${(\nu }_{1}$, $\nu_{2}$, $\nu_{4}{,\;\nu }_{5}{,\;\nu }_{6}{,\;\nu }_{7})$. In addition, the smart lamp (${\mathrm{SL}}_{j}$) sends information of the owner of the emergency signal patient ${\mathrm{Pa}}_{i}$ and $b_{i},$ so the medical institute (*MS*) is able to compute the verification parameter, $\nu_{3}$. Finally, the medical institute (*MS*) verifies the validity of the emergency signal by checking the equality between $\nu_{1}$ and ($\nu_{2}$, $\nu_{3}$, $\nu_{4}{,\;\nu }_{5}{,\;\nu }_{6}{,\;\nu }_{7}$) with $P_{\mathrm{MS}}$ and $P_{\mathrm{HA}}$. The process of verification can be referred to in [35], which has been proven.

(22) $$\nu_{1}{=T}_{\sigma_{{\mathrm{SL}}_{j}1}}(x)\;\mathrm{mod}\;p$$

(23) $$\nu_{2}{=T}_{H_{0}({\mathrm{PID}}_{i}{||\sigma }_{{\mathrm{SL}}_{j}2})}(x)\;\mathrm{mod}\;p$$

(24) $$\nu_{3}{=T}_{H_{1}({\mathrm{PID}}_{i}{||b}_{i})}(x)\;\mathrm{mod}\;p$$

(25) $$\nu_{4}{=T}_{H_{1}({\mathrm{EM}}_{i})}(x)\;\mathrm{mod}\;p$$

(26) $$\nu_{5}{=T}_{H_{0}({\mathrm{SLID}}_{j}{||\sigma }_{{\mathrm{SL}}_{j}3})}(x)\;\mathrm{mod}\;p$$

(27) $$\nu_{6}{=T}_{H_{1}({\mathrm{SLID}}_{j}{||b}_{j})}(x)\;\mathrm{mod}\;p$$

(28) $$\nu_{7}{=T}_{H_{2}({\mathrm{EM}}_{i})}(x)\;\mathrm{mod}\;p$$

(29) $$\nu_{1}{\;?=\nu }_{2}P_{\mathrm{MS}}\nu_{3}P_{\mathrm{HA}}\nu_{4}\sigma_{P_{i}2}\nu_{5}P_{\mathrm{MS}}\nu_{6}P_{\mathrm{HA}}\nu_{7}\sigma_{{\mathrm{SL}}_{j}3}$$

### 3.5. Secure Ambulance Communication Phase

**Figure 6:**
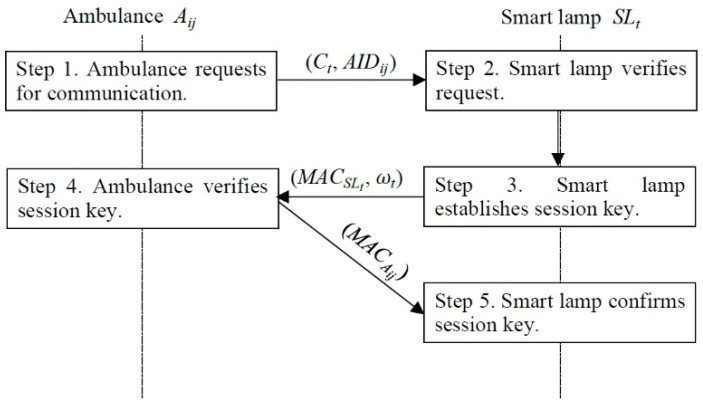
Secure ambulance communication phase.

After the ambulance ($A_{\mathrm{ij}}$) picks up the patient (${\mathrm{Pa}}_{i}$), the ambulance ($A_{\mathrm{ij}}$) can initiate communication with the server of the medical institute (*MS*) through the smart lamp (${\mathrm{SL}}_{t}$). The smart lamp (${\mathrm{SL}}_{t}$) and the ambulance ($A_{\mathrm{ij}}$) will execute mutual authentication to ensure further interaction between the smart lamp (${\mathrm{SL}}_{t}$) and the ambulance ($A_{\mathrm{ij}}$). Detailed descriptions are stated as follows and illustrated in Figure 6.

Step 1: The ambulance requests for communication. The ambulance ($A_{\mathrm{ij}}$) chooses a random number ($a_{\mathrm{ij}}$), computes $\mu_{\mathrm{ij}}$ and $C_{t}$, and sends ($C_{t}{,\;\mathrm{AID}}_{\mathrm{ij}}$) to the smart lamp (${\mathrm{SL}}_{t}$).

(30) $$\mu_{\mathrm{ij}}{=T}_{s_{\mathrm{ij}}}{(a}_{\mathrm{ij}})\;\mathrm{mod}\;p$$

(31) $$C_{t}{=(T}_{e_{t}}{(\mu }_{\mathrm{ij}}{||a}_{\mathrm{ij}}{||\mathrm{Certificate}}_{{\mathrm{SL}}_{j}\to A_{\mathrm{ij}}}{)\;\mathrm{mod}\;p)P}_{t}$$

Step 2: The smart lamp verifies the request. After receiving ($C_{t}{,\;\mathrm{AID}}_{\mathrm{ij}}$), the smart lamp (${\mathrm{SL}}_{t}$) obtains ${(\mu }_{\mathrm{ij}}{||a}_{\mathrm{ij}}{||\mathrm{Certificate}}_{{\mathrm{SL}}_{j}\to A_{\mathrm{ij}}})$ by decrypting $P_{t}$ and verifies if the ${\mathrm{Certificate}}_{{\mathrm{SL}}_{j}\to A_{\mathrm{ij}}}$ is valid. If the ${\mathrm{Certificate}}_{{\mathrm{SL}}_{j}\to A_{\mathrm{ij}}}$ is valid, the smart lamp (${\mathrm{SL}}_{t}$) progresses to the steps below, or the smart lamp (${\mathrm{SL}}_{t}$) abandons the request.

(32) $${(\mu }_{\mathrm{ij}}{||a}_{\mathrm{ij}}{||\mathrm{Certificate}}_{{\mathrm{SL}}_{j}\to A_{\mathrm{ij}}}{)=(T}_{d_{t}}{(C}_{t}{)\;\mathrm{mod}\;p)/P}_{t}$$

Step 3: The smart lamp establishes a session key. The smart lamp (${\mathrm{SL}}_{t}$) computes ($\omega_{t}$, ${\mathrm{sk}}_{{\mathrm{SL}}_{t}↔A_{\mathrm{ij}}}$, $P_{j}$, $P_{\mathrm{ij}}$, $P_{t}$, k, ${\mathrm{MAC}}_{{\mathrm{SL}}_{t}}$) and sends (${\mathrm{MAC}}_{{\mathrm{SL}}_{t}},\;\omega_{t}$) to the ambulance ($A_{\mathrm{ij}}$).

(33) $$\omega_{t}{=T}_{s_{t}}{(a}_{\mathrm{ij}})\;\mathrm{mod}\;p$$

(34) $${\mathrm{sk}}_{{\mathrm{SL}}_{t}↔A_{\mathrm{ij}}}{=H}_{2}{(T}_{s_{t}}{(\mu }_{\mathrm{ij}})\;\mathrm{mod}\;p)$$

(35) $$P_{j}{=H}_{1}({\mathrm{SLID}}_{j})$$

(36) $$P_{\mathrm{ij}}{=H}_{1}(W_{\mathrm{ij}}{,\;\mathrm{SLID}}_{j})$$

(37) $$P_{t}{=H}_{0}({\mathrm{SLID}}_{t})$$

(38) $$k=(P_{j}{||W}_{0})⊕(P_{\mathrm{ij}}{||W}_{i})⊕(P_{t}{||W}_{\mathrm{ij}})⊕({\mathrm{sk}}_{{\mathrm{SL}}_{t}↔A_{\mathrm{ij}}}\left|\right|\omega_{t})$$

(39) $${\mathrm{MAC}}_{{\mathrm{SL}}_{t}}{=h}_{k}{(P}_{t}{,\;P}_{\mathrm{ij}}{,\;\mu }_{\mathrm{ij}})$$

Step 4: The ambulance verifies the session key. After receiving (${\mathrm{MAC}}_{{\mathrm{SL}}_{t}},\;\omega_{t}$), the ambulance ($A_{\mathrm{ij}}$) computes (${\mathrm{sk}’}_{{\mathrm{SL}}_{t}↔A_{\mathrm{ij}}}$, k’) and verifies ${\mathrm{MAC}}_{{\mathrm{SL}}_{t}}$. If the result of the verification is true, the ambulance ($A_{\mathrm{ij}}$) computes ${\mathrm{MAC}}_{A_{\mathrm{ij}}}$ and sends ${\mathrm{MAC}}_{A_{\mathrm{ij}}}$ to the smart lamp (${\mathrm{SL}}_{t}$).

(40) $${\mathrm{sk}’}_{{\mathrm{SL}}_{t}↔A_{\mathrm{ij}}}{=H}_{2}(T_{s_{\mathrm{ij}}}(\omega_{t})\;\mathrm{mod}\;p)$$

(41) $$k’=(P_{j}{||W}_{0})⊕(P_{\mathrm{ij}}{||W}_{i})⊕(P_{t}{||W}_{\mathrm{ij}})⊕({\mathrm{sk}’}_{{\mathrm{SL}}_{t}↔A_{\mathrm{ij}}}\left|\right|\omega_{t})$$

(42) $$h_{k’}(P_{t}{,\;P}_{\mathrm{ij}}{,\;\mu }_{\mathrm{ij}})\;?={\mathrm{MAC}}_{{\mathrm{SL}}_{t}}$$

(43) $${\mathrm{MAC}}_{A_{\mathrm{ij}}}{=h}_{{\mathrm{sk}’}_{{\mathrm{SL}}_{t}↔A_{\mathrm{ij}}}}(P_{\mathrm{ij}}{,\;P}_{t},\;\omega_{t})$$

Step 5: The smart lamp confirms the session key. After receiving ${\mathrm{MAC}}_{A_{\mathrm{ij}}}$, the smart lamp (${\mathrm{SL}}_{t}$) verifies ${\mathrm{MAC}}_{A_{\mathrm{ij}}}$. If the result of the verification is true, a mutual authentication and key agreement is completed.

(44) $$h_{{\mathrm{sk}}_{{\mathrm{SL}}_{t}↔A_{\mathrm{ij}}}}{(P}_{\mathrm{ij}}{,\;P}_{t},\;\omega_{t}{)\;?=\mathrm{MAC}}_{A_{\mathrm{ij}}}$$

## 4. Security Analysis

This paper applies the random oracle model [36] and BAN logic [37] for formal security proof. The random oracle model [36] is used to prove the security of the emergency signal sending phase, and BAN logic [37] is used to prove the secure authentication of the secure ambulance communication phase. Note that the process of the random oracle model proof [36] can refer to other works using the random oracle model, including Liu’s work [38], because of a similar process of proof that aims to prove that the schemes can against eavesdropping attack to the Diffie–Hellman key exchange scheme. In addition, the process of BAN logic [37] can refer to other works using BAN logic, including Lee et al.’s [32] and Lin and Hsu’s [13] works, because of a similar process of proof that aims to prove that principals in schemes can believe established session keys. This paper will not describe the random oracle model and the BAN logic proof in detail. Informal security presents theoretical analyses that are present for proof of fulfillment of the security requirements of the proposed scheme.

### 4.1. Security of Secret Key

Assume an adversary wants to obtain the master secret key obtained by the server of the medical institute (*MS*), the smart lamp (${\mathrm{SL}}_{j}$), and the ambulance ($A_{\mathrm{ij}}$), such that $P_{\mathrm{MS}}=T_{s_{\mathrm{MS}}}(x)\;\mathrm{mod}\;p$ and $W_{j}=T_{s_{j}}(x)\;\mathrm{mod}\;p$. The adversary must have to solve the question based on CMDLP. If the adversary wants to obtain the smart lamp’s (${\mathrm{SL}}_{j}$’s) secret key, the adversary is required to solve the question based on CMDLP. On the other hand, the smart lamp (${\mathrm{SL}}_{j}$) generates the secret key for the ambulance ($A_{\mathrm{ij}}$) by performing $S_{\mathrm{ij}}=S_{j}T_{s_{j}}{(V}_{\mathrm{ij}})\;\mathrm{mod}\;p$. The smart lamp (${\mathrm{SL}}_{j}$) uses a private key ($S_{j}$) and a secret key ($s_{j}$) in the computing process, hence only the smart lamp (${\mathrm{SL}}_{j}$) is able to know the ambulance’s ($A_{\mathrm{ij}}$’s) secret key.

### 4.2. Key Confirmation and Security of Session Key

The ambulance ($A_{\mathrm{ij}}$) can check the session key (${\mathrm{sk}}_{{\mathrm{SL}}_{t}↔A_{\mathrm{ij}}}$) by ${\mathrm{MAC}}_{{\mathrm{SL}}_{t}}\;?=h_{k’}{(P}_{t}{,\;P}_{\mathrm{ij}}{,\;\mu }_{\mathrm{ij}})$, and the smart lamp (${\mathrm{SL}}_{t}$) can also check the session key (${\mathrm{sk}}_{{\mathrm{SL}}_{t}↔A_{\mathrm{ij}}}$) through ${\mathrm{MAC}}_{A_{\mathrm{ij}}}\;?=h_{{\mathrm{sk}}_{{\mathrm{SL}}_{t}↔A_{\mathrm{ij}}}}(P_{\mathrm{ij}}{,\;P}_{t},\;\omega_{t})$ in the proposed scheme. If the adversary wants to obtain the session key (${\mathrm{sk}}_{{\mathrm{SL}}_{t}↔A_{\mathrm{ij}}}$), the adversary has to solve CMDHP. Moreover, the session key (${\mathrm{sk}}_{{\mathrm{SL}}_{t}↔A_{\mathrm{ij}}}$) is not the same every time because of the random number ($a_{\mathrm{ij}}$). As a result, the proposed scheme achieves key confirmation while securing the session key.

### 4.3. Preventing Key-Compromise Impersonation Attacks

The ambulance’s ($A_{\mathrm{ij}}$’s) random number ($s_{\mathrm{ij}}$) can be stored in the onboard unit of the ambulance, which is hard to obtain information. On the other hand, the adversary cannot obtain k due to not knowing $s_{t}$, and afterwards, the process cannot be completed by the adversary. As a result, the proposed scheme can prevent key-compromise impersonation attacks.

### 4.4. Mutual Authentication

In the secure ambulance communication phase, the ambulance ($A_{\mathrm{ij}}$) and the smart lamp (${\mathrm{SL}}_{t}$) compute their session key k by public parameters (${\mathrm{SLID}}_{t}{,\;\mathrm{AID}}_{\mathrm{ij}}{,\;W}_{\mathrm{ij}}{,\;\mathrm{SLID}}_{j}$). In addition, each party generates a message authentication code (${\mathrm{MAC}}_{{\mathrm{SL}}_{t}}$) and ${\mathrm{MAC}}_{A_{\mathrm{ij}}}$ by k and ${\mathrm{sk}}_{{\mathrm{SL}}_{t}↔A_{\mathrm{ij}}}$ respectively to verify each other’s validity. Moreover, because of the feature of HIDC, the smart lamp (${\mathrm{SL}}_{t}$) can realize that the ambulance ($A_{\mathrm{ij}}$) comes from the cloud services provider by public parameter ${\mathrm{AID}}_{\mathrm{ij}}$.

### 4.5. Preventing MITM Attack

In order to prevent an MITM attack in the secure ambulance communication phase, the ambulance ($A_{\mathrm{ij}}$) and the smart lamp (${\mathrm{SL}}_{t}$) can confirm whether the message is resent, modified, and replaced, by checking the information through message authentication codes ${\mathrm{MAC}}_{{\mathrm{SL}}_{t}}$ and ${\mathrm{MAC}}_{A_{\mathrm{ij}}}$. This means that the adversary cannot modify the message authentication codes ${\mathrm{MAC}}_{{\mathrm{SL}}_{t}}$ and ${\mathrm{MAC}}_{A_{\mathrm{ij}}}$ without the session key ${\mathrm{sk}}_{{\mathrm{SL}}_{t}↔A_{\mathrm{ij}}}$. Thus, the proposed scheme can prevent an MITM attack.

### 4.6. Unforgeability

If the adversary wants to forge a validated anonymous identity, the adversary has to acquire smart lamp’s (${\mathrm{SL}}_{j}$’s) secret ($s_{j}$) and private key ($S_{j}$). The adversary has to solve CMDLP if the adversary wants to compute the smart lamp’s (${\mathrm{SL}}_{j}$’s) secret ($s_{j}$) and private key ($S_{j}$) from public parameter ($W_{j}$).

### 4.7. Without Assistance of Registration Center

The registration center (RC) is a third party for both sides of communication after the registration phase. A privilege or malicious insider attack may occur if the adversary is in the RC, and some risks may be led to, such as message leakage, verifications stolen, etc. If a privilege or malicious insider attack occurs in a telemedicine system, the patient’s privacy and security may be damaged. Although works related to the security of the 5G networks have been proposed recently [3,4], the RC is included in the system structure of these works, which is no different from conventional networks. In the proposed scheme, the hierarchical system structure was introduced, which is suitable for 5G networks without a RC or a trusted third party.

### 4.8. Resistant to Bergamo et al.’s Attack 

Bergamo et al. proposed an attack on Chebyshev chaotic maps-based cryptosystems based on two reasons as below [39]. First, an adversary is able to obtain related elements (*x*, $a_{\mathrm{ij}}$, $\mu_{\mathrm{ij}}$, $\omega_{j}$). Second, several Chebyshev polynomials go through the same point due to the periodicity of the cosine function. In the proposed scheme, an adversary is unable to obtain any related elements (*x*, $a_{\mathrm{ij}}$, $\mu_{\mathrm{ij}}$, $\omega_{j}$) because of being encrypted in transmitted messages where only the ambulance ($A_{\mathrm{ij}}$) and the smart lamp (${\mathrm{SL}}_{j}$) can retrieve the decryption key. Moreover, the proposed scheme utilizes extended Chebyshev polynomials proposed by Zhang [34], in which the periodicity of the cosine function can be avoided. As a result, the proposed scheme can resist attack proposed by Bergamo et al. [39].

## 5. Computational Complexity Analysis

According to previous research that uses MIRACL Library and Ubuntu 16.0 operating system with 4 GB RAM and 2.7 GHz processor and get execution time [3,4,13], the time of performing a one-way hash function operation ($T_{h}$) is about 0.006 milliseconds (ms), and time for performing a Chebyshev chaotic maps operation ($T_{\mathrm{ch}}$) is approximately equal with 42.04 times of performing a one-way hash function operation that is about 0.252 ms and using Chebyshev chaotic maps can be more efficient than using elliptic-curve cryptography. The time taken for computing XOR operations is ignored because the value is too low to influence the result. The results of computational complexity and performing time of the proposed scheme are presented and shown in Table 3. In the emergency signal sending phase, the patient will take 0.006 ms, the smart lamp will take 0.012 ms, and the server of the medical institute will take 1.8 ms after receiving a message from the patient. The ambulance does not exist in the emergency signal sending phase. Performing the emergency signal sending phase will take at least 1.818 ms, according to the results above. In the secure ambulance communication phase, the ambulance will take 0.792 ms, and each smart lamp will take 0.774 ms after receiving a message from the ambulance. The patient and server of the medical institute do not exist in the secure ambulance communication phase. Performing the secure ambulance communication phase will take at least 1.566 ms, according to the results above. Although there are no requirements or standards about the recommendation of time to perform a cryptographic module, the proposed scheme has proven that is more efficient than the previous studies. For example, the time to perform the emergency signal sending phase is better than Abdel-Malek et al.’s work [40]; the process of the secure ambulance communication phase is similar to Lin and Hsu’s [13] work so that the results can be referred to Lin and Hsu’s [13] work.

## 6. Conclusions

5G networks provide high-speed network, big capacity, and scalability, which has an efficient effect on energy consumption and provides quality of experience and amount of devices communication, and 5G can provide connection massive IoT. IoT with 5G environments provides solutions of the network layer, including enhancing the quality of service, to solve challenges of smart medical healthcare. EMS has become a complex network of all types of professionals, including care in an ambulance. 5G network with EMS can simplify the medical treatment process and improve the efficiency of patient treatment. The importance of healthcare-related privacy preservation is rising. If the work of privacy preservation fails, not only will medical institutes have economic and credibility losses but also property losses and even the lives of patients will be harmed. This paper proposes a privacy-preserved ID-based secure communication scheme in 5G-IoT telemedicine systems that can achieve the features below. The proposed scheme allows the emergency signal to be transmitted immediately with decreasing risk of secret key leakage. Information about the patient and their prehospital treatments can be transmitted securely while transferring the patient to the destination medical institute, and the quality of healthcare services can be assured while preserving the privacy of the patient through the proposed scheme. The proposed scheme supports not only normal situations but also emergencies. The proposed scheme applies key insulation to prevent key exposure problems on wearable devices and provides federated identity management, which can manage the identity of ambulances in a hierarchical structure efficiently. Finally, the proposed scheme can resist potential attacks and has been proven secure enough using the random oracle model [36] and BAN logic [37].

## Figures and Tables

**Table 1:** Mathematical definitions of Chebyshev chaotic maps.

Mathematical Definitions	Descriptions
Chebyshev polynomial	$\mathrm{Chebyshev}\;\mathrm{polynomial}\;T_{n}(x):\;\to [-1,1]$ is a polynomial in *x* of degree *n*, defined as $T_{n}{(x)=\cos(\mathrm{ncos}}^{-1}\left(x)\right)$.
Recurrent relation	$T_{n}{(x)=2xT}_{n-1}{(x)-T}_{n-2}\left(x\right)$for any n ≥ 2$,\;T_{0}(x)=1$$,\;\mathrm{and}\;T_{1}(x)=x$.
Semi-group property	$T_{r}{(T}_{s}{(x))=T}_{rs}{(x)=T}_{s}{(T}_{r}\left(x)\right)$for any (s, r)∈Zand s∈[−1,1]. Chebyshev polynomial restricted to interval [–1, 1] is a well-known chaotic map for all *n* > 1, which has a unique continuous invariant measure with positive Lyapunov exponent ln *n*. For *n* = 2, Chebyshev maps reduces to well-known logistic maps.
Extended Chebyshev polynomials	Zhang [34] proved that the semi-group property holds for Chebyshev polynomials defined on interval (−∞,+∞), and extended Chebyshev polynomials is defined as $T_{n}{(x)=(2\mathrm{xT}}_{n-1}{(x)-T}_{n-2}(x))\;\mathrm{mod}\;N$, where n ≥ 2, x∈(−∞,+∞), and *N* is a large prime number. Semi-group property holds, and extended Chebyshev polynomials also commute as $T_{r}(T_{s}{(x))\;\mathrm{mod}\;N=T}_{rs}{(x)\;\mathrm{mod}\;N=T}_{s}(T_{r}(x))\;\mathrm{mod}\;N$.
Chaotic maps-based discrete logarithm problem (CMDLP)	Given two elements *x* and *y*, it is computationally infeasible to find the integer *n* such that $T_{n}(x)\;\mathrm{mod}\;N=y$.
Chaotic maps-based Diffie-Hellman problem (CMDHP)	$\mathrm{Given}\;\mathrm{three}\;\mathrm{elements}\;x,\;T_{r}(x)\;\mathrm{mod}\;N$$,\;\mathrm{and}\;T_{s}(x)\;\mathrm{mod}\;N$$,\;\mathrm{it}\;\mathrm{is}\;\mathrm{computationally}\;\mathrm{infeasible}\;\mathrm{to}\;\mathrm{compute}\;T_{rs}(x)\;\mathrm{mod}\;N$.

**Table 2:** Notations of proposed scheme.

Notations	Definitions
${PID}_{i}$	$\mathrm{Identity}\;\mathrm{of}\;\mathrm{patient}\;{Pa}_{i}$
${SLID}_{j}$	$\mathrm{Identity}\;\mathrm{of}\;\mathrm{smart}\;\mathrm{lamp}\;{SL}_{j}$.
${AID}_{ij}$	$\mathrm{Identity}\;\mathrm{of}\;\mathrm{ambulance}\;A_{ij}$.
*k*	Encryption/decryption key *k*.
$E_{k}$$(.)/D_{k}$(.)	A symmetric encryption/decryption algorithm with secret key *k*.
$S_{j}$	$\mathrm{Private}\;\mathrm{key}\;\mathrm{of}\;\mathrm{smart}\;\mathrm{lamp}\;{SL}_{j}$.
$S_{ij}$	$\mathrm{Private}\;\mathrm{key}\;\mathrm{of}\;\mathrm{ambulance}\;A_{ij}$.
${sk}_{{SL}_{j}↔A_{ij}}$	$\mathrm{Session}\;\mathrm{key}\;\mathrm{of}\;\mathrm{smart}\;\mathrm{lamp}\;{SL}_{j}$$\;\mathrm{and}\;\mathrm{ambulance}\;A_{ij}$.
$p,\;p_{j}$ $,\;q_{j}$	Large random prime numbers.
$x,\;e_{j}$ $,\;d_{j}$	Random numbers.
$h_{k}$(.)	Collision-resistance secure one-way keyed chaotic hash function.
$s_{MS}{,\;\omega }_{MS}$	The secrete values of server of medical institute (*MS*).
⊕	Exclusive OR (XOR) operation.
*A* ?= *B*	Checking if value *A* is equal to *B* or not.
${MAC}_{A}$	The message authentication code algorithm of *A*.
${\mathrm{Certificate}}_{HCA\to \mathrm{MS}}$	Certification issued by healthcare certification authority to a server of a medical institute *(MS).*
${\mathrm{Certificate}}_{\mathrm{MS}\to {\mathrm{SL}}_{j}}$	$\mathrm{Certification}\;\mathrm{issued}\;\mathrm{by}\;a\;\mathrm{server}\;\mathrm{of}\;a\;\mathrm{medical}\;\mathrm{institute}\;(\mathrm{MS})\;\mathrm{to}\;a\;\mathrm{smart}\;\mathrm{lamp}\;{\mathrm{SL}}_{j}$$\mathrm{that}\;\mathrm{is}\;\mathrm{generated}\;\mathrm{from}\;{\mathrm{Certificate}}_{\mathrm{HCA}\to \mathrm{MS}}$.
${\mathrm{Certificate}}_{{\mathrm{SL}}_{j}\to A_{\mathrm{ij}}}$	$\mathrm{Certification}\;\mathrm{issued}\;\mathrm{by}\;a\;\mathrm{smart}\;\mathrm{lamp}\;{\mathrm{SL}}_{j}$$\;\mathrm{to}\;\mathrm{an}\;\mathrm{ambulance}\;A_{\mathrm{ij}}$$\mathrm{that}\;\mathrm{is}\;\mathrm{generated}\;\mathrm{from}\;{\mathrm{Certificate}}_{\mathrm{MS}\to {\mathrm{SL}}_{j}}$.
*w*	Warrant including delegation information.
$b_{i}$ $,\;b_{s}$	Number of key update time.
${\mathrm{EM}}_{i}$	Emergency signal.

**Table 3:** Performance analysis of proposed scheme.

	Phase	Emergency Signal Sending Phase	Secure Ambulance Communication Phase
Role			
$\mathrm{Patient}\;{\mathrm{Pa}}_{i}$		$T_{h}=0.006\mathrm{ms}$	N/A
$\mathrm{Smart}\;\mathrm{lamp}\;{\mathrm{SL}}_{j}$		${2T}_{h}=0.012\mathrm{ms}$	${3T}_{\mathrm{ch}}{+6T}_{h}=\left(0.756+0.036\right)\;\mathrm{ms}=0.792\;\mathrm{ms}$
Server of medical institute *MS*		${7T}_{\mathrm{ch}}{+6T}_{h}=\left(0.036+1.764\right)\;\mathrm{ms}=1.8\;\mathrm{ms}$	N/A
$\mathrm{Ambulance}\;A_{\mathrm{ij}}$		N/A	${3T}_{\mathrm{ch}}{+3T}_{h}=\left(0.756+0.018\right)\;\mathrm{ms}=0.774\;\mathrm{ms}$
Total		${7T}_{\mathrm{ch}}{+9T}_{h}=\left(0.054+1.764\right)\;\mathrm{ms}=1.818\;\mathrm{ms}$	${6T}_{\mathrm{ch}}{+9T}_{h}=\left(1.512+0.054\right)\;\mathrm{ms}=1.566\;\mathrm{ms}$

## Data Availability

Not applicable.
